# Exploring the immediate and short-term effect of lumbar spinal manipulation on pressure pain threshold: a randomized controlled trial of healthy participants

**DOI:** 10.1186/s12998-024-00540-5

**Published:** 2024-05-29

**Authors:** Matthew R. Schumacher, Colton Swanson, Saydee Wolff, Rylee Orteza, Rudy Aguilar

**Affiliations:** University of Mary, Bismarck, ND USA

**Keywords:** Lumbar manipulation, Pain pressure threshold, Neurophysiological effects, Manual therapy

## Abstract

**Background:**

Lumbar spinal manipulative therapy (SMT) is a common intervention used to treat low back pain (LBP); however, the exact neurophysiological mechanisms of SMT reducing pain measured through pain pressure threshold (PPT) have not been fully explored beyond an immediate timeframe (e.g., immediately or five-minutes following) referencing a control group. Therefore, the purpose of this study was to investigate the neurophysiological effects of lumbar SMT compared to deactivated ultrasound using PPT immediately following and 30-minutes following SMT.

**Methods:**

A longitudinal, randomized controlled trial design was conducted between September to October 2023. Fifty-five participants were randomized into a control group of deactivated ultrasound (*n* = 29) or treatment group of right sidelying lumbar SMT (*n* = 26). PPT, recorded at the right posterior superior iliac spine (PSIS), was documented for each participant in each group prior to intervention, immediately, and 30-minutes after. A repeated measures ANOVA, with a post-hoc Bonferroni adjustment, was used to assess within-group and between-group differences in PPT. The significance level was set at a < 0.05 a priori.

**Results:**

Statistically significant differences were found between the deactivated ultrasound and lumbar SMT groups immediately (*p =* .05) and 30-minutes (*p =* .02) following intervention. A significant difference in the lumbar SMT group was identified from baseline to immediately following (*p <* .001) and 30-minutes following (*p <* .001), but no differences between immediately following and 30-minutes following intervention (*p =* .10). The deactivated ultrasound group demonstrated a difference between baseline and immediately after intervention with a reduced PPT *(p =* .003), but no significant difference was found from baseline to 30-minutes (*p =* .11) or immediately after intervention to 30-minutes (*p =* 1.0).

**Conclusion:**

A right sidelying lumbar manipulation increased PPT at the right PSIS immediately after that lasted to 30-minutes when compared to a deactivated ultrasound control group. Future studies should further explore beyond the immediate and short-term neurophysiological effects of lumbar SMT to validate these findings.

**Trial Registration:**

This study was retrospectively registered on 4 December 2023 in ClinicalTrials (database registration number NCT06156605).

## Introduction

Lumbar spinal manipulation therapy (SMT) is a common intervention used to treat low back pain (LBP) [1–3], defined as a high-velocity, low-amplitude technique (HVLAT) performed at the pathological limit of the joint [4, 5]. Several theories have been reported regarding the various mechanisms of lumbar SMT, including the vertebral subluxation theory suggesting malignments and biomechanical faults corrected through the use of SMT [6, 7]. Although SMT has been shown to be effective in reducing pain and disability in acute and chronic lumbar conditions [2, 8], the exact mechanism of SMT continues to be explored [9, 10]. 

Many studies have supported the theory of neurophysiological and biomechanical mechanisms of SMT [11–15]. For example, Fritz et al. [14] explored the effects of SMT on lumbar spinal stiffness, lumbar multifidus recruitment, and status on a clinical prediction rule for SMT outcomes. SMT mechanisms were discovered to be multi-factorial with significant changes in spinal stiffness and multifidus recruitment [14]. Additionally, Bialosky et al. provided a framework on the individual neurophysiological mechanisms that occur from SMT within the tissue, peripheral nervous system, spinal cord, and higher level centers of the brain [16, 17]. Peripherally, it has been theorized that SMT has localized tissue effects involving a reduction of local inflammatory factors that facilitate local muscular control [16, 17]. It has also been shown that the effects of SMT may have a more centralized origin proposing significant central nervous system mechanisms including the somatosensory cortex, amygdala, anterior cingulate cortex, periaqueductal gray, and rostral ventromedial medulla [16–18]. As such, one common and simple method of measurement to assess for this quantitative pain sensitivity at a particular site is pain pressure threshold (PPT), defined as the minimum amount of pressure using an algometer that can be applied before inducing pain or discomfort [19, 20]. 

Most research regarding this topic has investigated the immediate effects of lumbar SMT using PPT with limited research exploring beyond an immediate timeframe [20–23]. For example, a systematic review by Honoré et al. [24] highlighted the neurophysiological effects of PPT being the greatest at five-minutes following that minimized to ten-minutes post-lumbar SMT in asymptomatic participants, but did not explore beyond this timeframe. Dorron et al. [25] also explored PPT beyond the immediate effects of lumbar SMT up to 30-minutes, however, lacked a true control group by comparing between a right and left side lumbar manipulation.

Given this lack of understanding of the neurophysiological effects of lumbar SMT beyond an immediate follow-up with a control group comparison, the aim of this study was to investigate PPT beyond the immediate effect up to 30-minutes post-lumbar SMT using a deactivated ultrasound control. We hypothesized there would be a difference in the experimental and control group at 30-minutes following each intervention. Analyzing the effect of lumbar SMT using PPT 30-minutes following may provide greater insight on the true neurophysiological effects of lumbar SMT.

## Methods

### Study design

This study was a longitudinal, randomized controlled trial that took place on the campus of University of Mary, Bismarck, North Dakota, United States of America from September to October 2023. The project was approved by the institution’s institutional review board ([IRB] Project 2,260,080,323) and was registered retrospectively on clinicaltrials.gov (NCT06156605).

### Recruitment

Participants, including faculty, staff, and students on the University of Mary campus, were recruited via convenience sampling through email, flyers, and word-of-mouth. To be included in this study, participants had to be asymptomatic for LBP and between the ages of 18–50. Participants were excluded if they had: (1) a history of LBP within the last three months, (2) any previous spinal surgeries, (3) any rheumatological condition or neurological symptoms/conditions, (4) a recent ingestion of pain-relieving medications within the last 24 h, (5) a lumbar manipulation within three days prior, or (6) exhibited any contraindications that would preclude them from receiving lumbar manipulation as assessed through a health history questionnaire.

### Outcome variable

PPT was measured before and after each intervention at the right posterior superior iliac spine (PSIS). This method has been reported as valid and reliable in comparison to a gold standard force platform [26]. The instrument used to assess PPT was the Wagner Pain Test FPX**™** 50 algometer (Wagner Instruments, Riverside, Connecticut, United States of America), by the same method as previous studies assessing PPT [27, 28]. 

### Randomization

Participants who met the inclusion criteria were invited to take part in the study. Those who conveyed interest were informed of the purpose with an explanation of any risks from partaking in the study, per the University of Mary IRB committee approval. Each participant provided informed consent before enrolling in the study. If eligible, participants were randomized through a simple block randomization process using a standard deck of 52 cards by a separate research member (SW). The cards were shuffled and stacked in a single pile face down when they were selected, randomized by either a red or black card. For the remaining three participants, the cards were reshuffled. Each participant’s confidentiality throughout randomization was kept through a concealed coding process for privacy considerations until interventions were assigned. Each participant was randomized to either the experimental group of lumbar SMT or control group of deactivated ultrasound. Participants within the study were not blinded to the other intervention group. Participants in the deactivated ultrasound group were aware of the intervention; however, were blinded to the fact that ultrasound was deactivated, e.g., a placebo. The outcome assessor (CS) measuring PPT was blinded to the randomization process; however, given the nature of the study, the participants and research members providing treatment were not blinded to the treatment provided in each arm of the study.

### Assessment

**Fig. 1:**
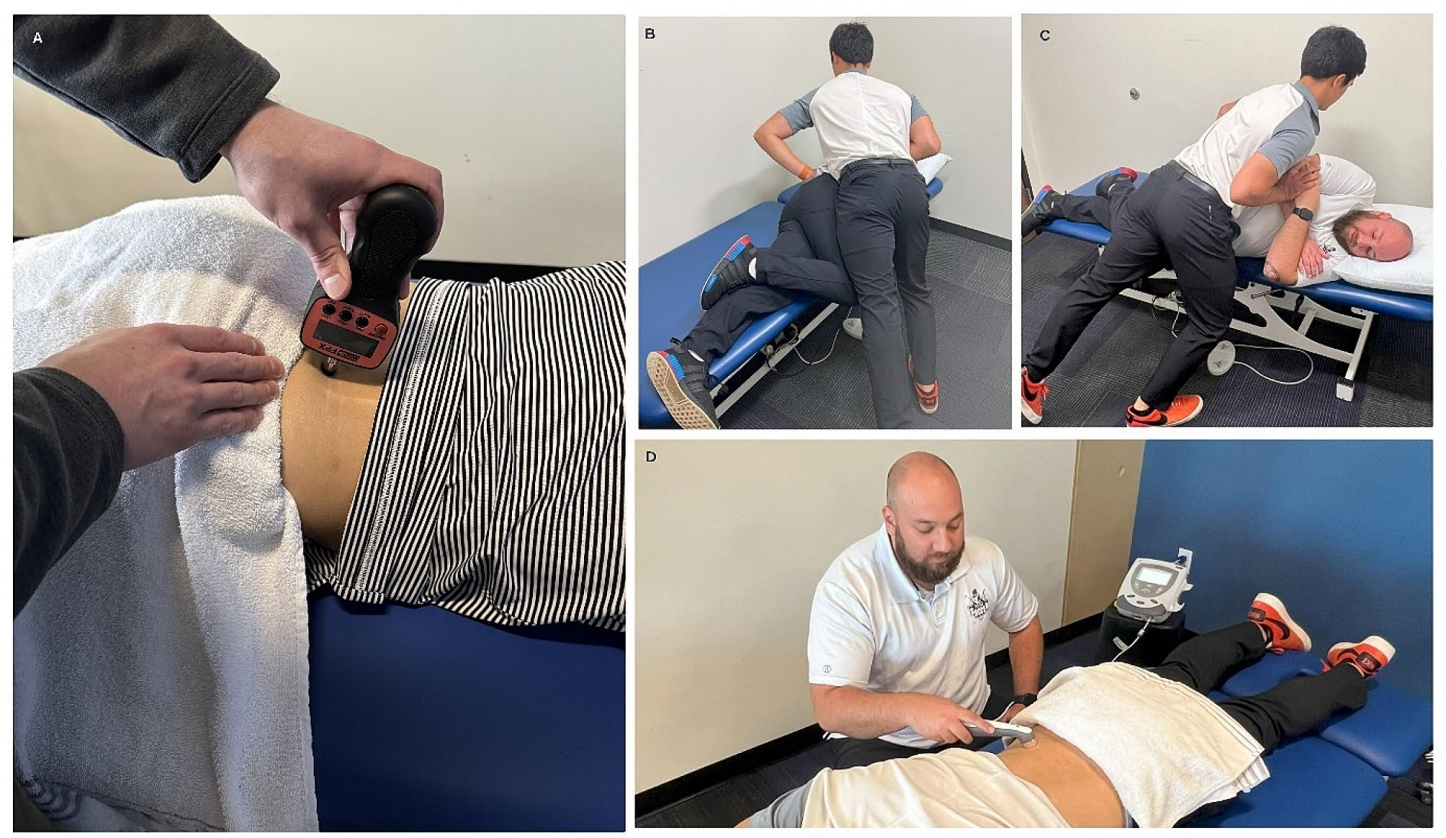
(**A**) Assessment of pain pressure threshhold at the posterior superior iliac spine using the algometer, Interventions performed with patient positioning for the (**B**) sidelying lumbar manipulation from the side, (**C**) sidelying lumbar manipulation from a posterior view, and (**D**) deactivated ultrasound to the posterior superior iliac spine.

Following inclusion, each participant underwent a standardized PPT assessment using an algometer prior to each intervention by one outcome assessor. The algometer was placed perpendicular to the skin at the site of the PSIS on the right side after being properly identified (Fig. 1). The outcome assessor completed all measurements for all time points to ensure reliability and consistency with all measurements. After a thorough description of the assessment, each participant was instructed to say stop when the sensation of pressure changed to feeling unpleasant, at which it was removed, and the digital pressure reading was recorded within two decimal places. This was completed once per measurement per time point. PPT testing was completed before the intervention, immediately post-intervention, and 30-minutes post-intervention. In addition, participants were instructed to remain in the near vicinity with instructions not to exercise or receive any form of manual therapy between assessment timepoints.

### Interventions

Following randomization and assessment, each participant was allocated to their respective intervention group. There were two independent variables including treatment allocation (levels: lumbar SMT and deactivated ultrasound) and time following treatment (levels: before, immediately after, and 30-minutes after treatment). Lumbar SMT was performed by two research members (MS, RO) targeting the L5-S1 segment. The first research member (RO) had less than one year of experience and the other (MS) had eight years of experience and post-professional orthopaedic manual therapy training. Each participant was instructed to a left sidelying position. Once set up, a consistent description of the intervention was provided for each participant. In this left sidelying position, the lower leg was positioned straight, while the top knee and hip were flexed. The trunk was rotated down to where the research members felt they were able to bias the L5-S1 segment using the mammillary push lateral recumbent position. The participant’s arms were positioned in the front of the abdomen where the research member’s right hand made contact to block the participant’s upper trunk from moving during the technique. The research member’s left hand was placed on the right posterior hip where the lower trunk was rotated forward to approximately 45 degrees from start position until slight tension was felt. A HVLAT was applied through the research member’s body to the participant’s right side (Fig. 1). This was delivered once regardless of an audible popping sound being heard or not.

Deactivated ultrasound was administered by a different research member (RA) consistently at a deactivated setting of a duty cycle of 100%, frequency of three megahertz, intensity of zero watts per centimeter squared with a five centimeter sound head for a duration of two and half minutes [29]. Each participant was positioned prone where the deactivated ultrasound was applied to their right lumbar region between the ribcage and pelvis at the right PSIS with a consistent description of the intervention (Fig. 1). Although the participants were not blinded to the other intervention group, each participant in this control group was blinded to the fact that the ultrasound was set at deactivated parameters.

### Statistical analysis

G*Power 3.1 software was used to calculate a sample size a priori, determining the need for at least 12 participants per group for total of 24 participants using a medium effect size (0.5) with 80% power and alpha level of 0.05 with three degrees of freedom. This was determined to be sufficient if the allocation ratio was not one-to-one.

All statistical analyses were completed with SPSS 28.0 (IBM, Armonk, New York, United States of America). A normal distribution was established using the Shapiro-Wilk test (*p* > .05) and visual inspection using histogram and Q-Q plot. All descriptive data was reported for continuous data using means and standard deviations (age, baseline PPT scores) and categorical data (gender).

A repeated measures analysis of variance (ANOVA) was performed to assess intervention, time, and time*intervention interaction effects. All assumptions were analyzed including testing the outcome variable for normality using the Shapiro-Wilk test, assessment of homogeneity of variance using the Lavene’s test of equality, assessment of multicollinearity using tolerance values and variance inflation factor, assessment of normal distribution of residuals using a scatter plot, and assessment of sphericity using the Mauchly’s test of sphericity. The within-group effect size was calculated using a partial eta squared (ηp^2^) value. The alpha level was set at *p* < .05 a priori.

A post-hoc analysis Bonferroni adjustment was run to assess a pairwise comparison of PPT scores of time*intervention immediately and 30-minutes following. This was also run to assess groupwise comparison to analyze the subsets of PPT scores to determine significant differences within each group over time, including baseline, immediately following, and 30-minutes following.

## Results

**Fig. 2:**
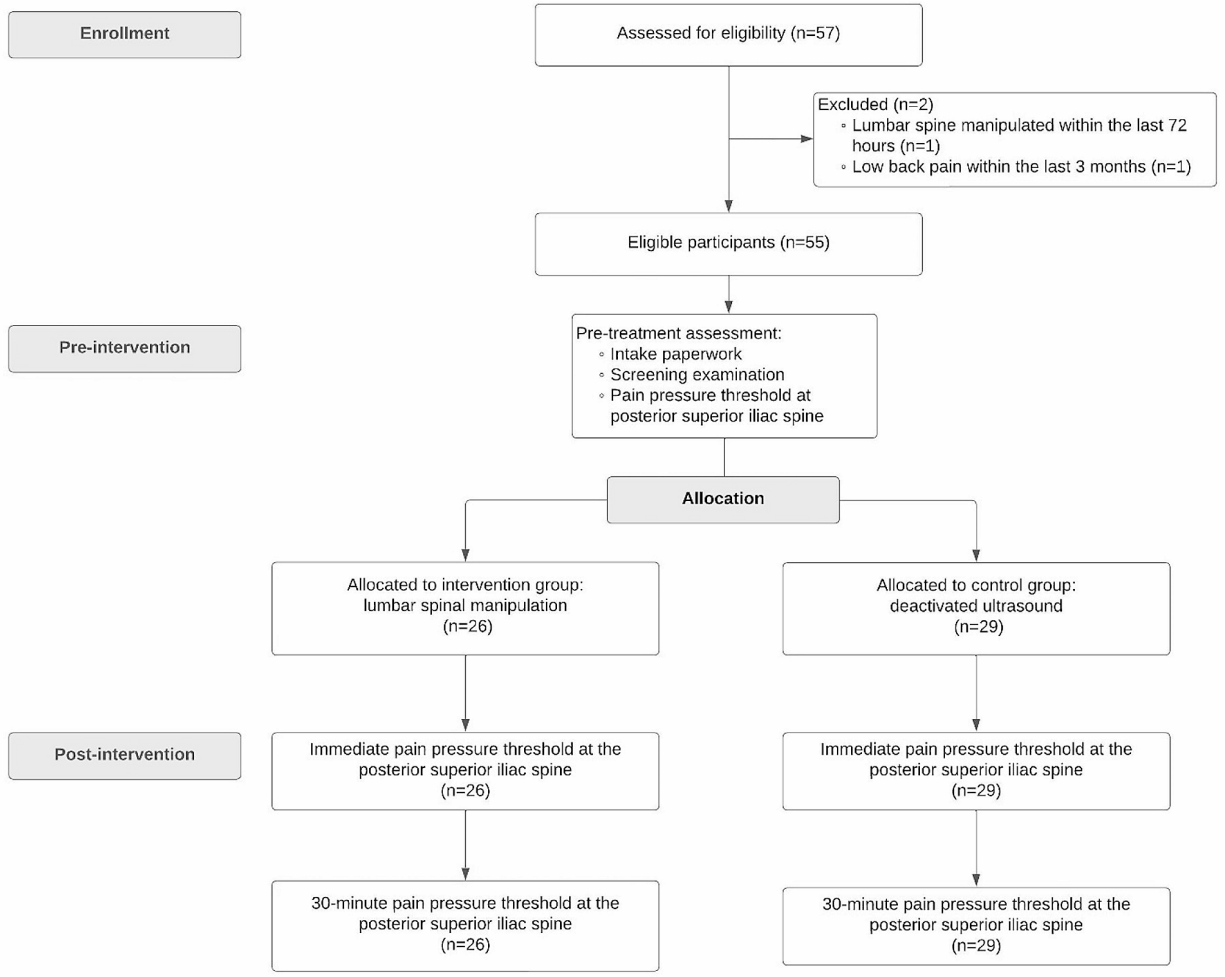
CONSORT flow diagram of the participants

A total of 57 participants were screened for eligibility to be included within the study. Two participants were excluded as per the inclusion and exclusion criteria (Fig. 2). Fifty-five participants, including 35 females (63.6%) and 20 males (36.4%), between the ages of 18–50 met the inclusion criteria and were included in the trial. The average age for those in the lumbar SMT group was 24.45 years (standard deviation = 4.42) and deactivated ultrasound was 23.45 years (standard deviation = 1.86). See Table 1 for a descriptive summary of the participant characteristics including age, gender, and baseline PPT scores. A simple block randomization process of the 55 participants resulted in 29 individuals (16 females; 13 males) into the deactivated ultrasound group and 26 individuals (19 females; 7 males) into the sidelying lumbar SMT treatment group. No patients were lost to follow-up at any time point, and no adverse events were reported during the trial in either group.

**Table 1:** Baseline characteristics of participants

Groups	Lumbar SMT	Deactivated ultrasound	*P* value
Age in years (mean, SD)	24.5, 4.42	23.45, 1.86	*p* = .30
Gender (n)	26 (19 F, 7 M)	29 (16 F, 13 M)	
Immediate PPT in lbs (mean, SD)	10.18, 3.63	10.68, 3.32	*P* = .60

Abbreviations: PPT = pain pressure threshold, n = sample size, lbs. = pounds, SD = standard deviation, F = female, M = male

PPT scores at baseline were found to be normally distributed using the Shapiro-Wilk Test (*p* = .20). All assumptions for the repeated measures ANOVA were met assessing homogeneity of variance using Lavene’s test of equality at baseline (*p* = .98), immediately following (*p* = .73), and 30-minutes following (*p* = .16), assessment of multicollinearity using tolerance values and variance inflation factor, and sphericity using Mauchley’s test of sphericity (*p* = .09) were met.

No significant differences in PPT scores at all timepoints were found between groups (F = 1.86, *df* = 1, *p* = .18, ηp^2^ = 0.03) (Table 2); however, a post-hoc analysis determined significance between treatment groups immediately after intervention (*p* = .05, 95% CI -3.72, 0.008) and 30-minutes after intervention (*p =* .02, 95% CI -4.04, -0.46) (Table 3).

**Table 2:** Tests of within-subjects and between-subjects for pain pressure threshold measurement

Source	Type III Sum of Squares	df	F value	*P* value	Partial eta squared value
**Within-Subjects**					
Time					
Sphericity assumed	11.07	2	3.81	0.03	0.07
Time*Intervention					
Sphericity assumed	60.66	2	20.88	< 0.001	0.28
**Between-subjects**					
Intervention	59.39	1	1.86	0.18	0.03

*Abbreviations: df = degrees of freedom*

**Table 3:** Post-hoc Bonferroni analysis for within-group and between-group mean differences with *p* values and 95% confidence intervals

	Within-group	Between-group		
**Lumbar SMT** *n* = 26	Within-group mean difference (lbs.), 95% CI, *p* value	Between-group mean difference, 95% CI, *p* value		
Baseline to immediately following	-1.32 (-2.11, -0.54), *p* < .001	Baseline	0.50 (-1.38, 2.38), *p* = .60	
Baseline to 30-minutes following	-1.98 (-2.92, -1.05), *p* < .001	Immediately following SMT	-1.86 (-3.72, 0.01), *p* = .05	
Immediately following to 30-minutes following	-0.66 (-1.40, 0.08), *p* = .10	30-minutes following SMT	-2.25 (-4.04, -0.46), *p* = .02	
**Deactivated ultrasound** *n* = 29	Within-group mean change, 95% CI, F value, and effect size			
Baseline to immediately following	1.03 (0.29, 1.77), *p* = .003			
Baseline to 30-minutes following	0.78 (-0.12, 1.66), *p* = .11			
Immediately following to 30-minutes following	0.27 (-0.44, 0.97), *p* = 1.0			

*Abbreviations*: CI = confidence interval, lbs. = pounds, SMT = spinal manipulative therapy

**Fig. 3:**
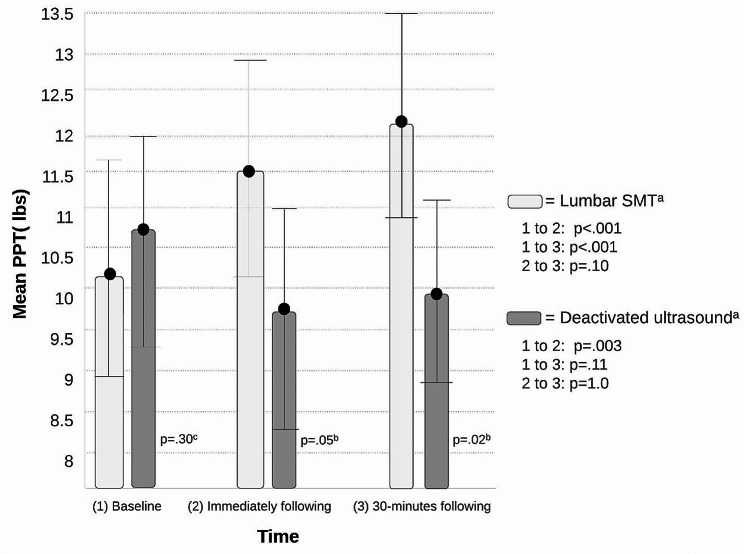
Mean pain pressure threshold by group and time. Abbreviations: PPT: Pain pressure threshold; lbs: pounds. a = Post hoc analysis with a Bonferroni adjustment between different time points. b = Repeated measures ANOVA between groups at different time points. c = Independent samples T-test for baseline mean differences between groups

A significant time (F = 3.81, *df* = 2, *p =* .03, ηp^2^ = 0.07) and time*intervention (F = 20.88, *df* = 2, *p <* .001, ηp^2^ = 0.28) interaction for PPT values were identified (Table 2). A post-hoc analysis found statistically significant differences in the lumbar SMT group from baseline to immediately following (*p <* .001, 95% CI -2.11, -0.54) and 30-minutes following (*p <* .001, 95% CI -2.92, -1.05), but no differences between immediately after and 30-minutes following intervention (*p =* .10, 95% CI -1.40, 0.08) (Table 3). The deactivated ultrasound group demonstrated a difference between baseline and immediately following *(p =* .003, 95% CI 0.29, 1.77) with a decreased PPT, but no significant difference was found from baseline (*p =* .11, 95% CI -0.12, 1.66) or immediately after intervention (*p =* 1.0, 95% CI -0.97, 0.44) to 30-minutes. See Fig. 3 for mean PPT scores over time.

## Discussion

To the author’s knowledge, this is the first trial to highlight the lumbar SMT hypoalgesia effects on mechanical PPT beyond an immediate timeframe referencing a true control group [20–24, 30–32]. Participants in the lumbar SMT were found to experience a greater threshold of pain at the right side PSIS over a 30-minute timeframe that was not found in the deactivated ultrasound group, consistent with the researcher’s hypothesis that there would be a difference between-groups and within-group means at each timepoint.

The initial increase in PPT observed both immediately and 30-minutes post SMT is consistent with the findings from the reviews of Honoré et al. [33] and Coronado et al. [34], which established a medium-to-large effect size during the first 5-minutes post treatment. Similar results demonstrating the immediate increase in PPT have been observed in studies where SMT was applied to the cervical spine [20, 22]. The effect of SMT on the thoracic spine using PPT has been less established with results by Honoré et al. [33, 35] showing no regional or remote effects to PPT when compared to a valid sham procedure. Additionally, Dorron et al. [25] reported similar findings to this study in that lumbar SMT had a lasting effect of 30-minutes via PPT; however, a control group was not used only comparing left side lumbar SMT to right side lumbar SMT. Additionally, PPT using multiple sites distal to the site of the SMT showed a lasting increase in PPT, while the sites proximally did not [25]. Although we did not investigate PPT sites distal to the SMT, these findings were consistent with the results of our study with local (L5-S1) changes at the PSIS where SMT was performed. Interestingly, we also found that PPT statistically decreased in the deactivated ultrasound group from baseline to immediately and 30-minutes following. One possible explanation for this may have been an increase in skin sensitivity with no neurological hypoalgesia benefits like that seen in SMT group. However, we recommend further research to explore these findings.

Understanding the concept of the hypoalgesia effects of lumbar SMT is clinically relevant, particularly regarding the timeframe of effects following this intervention. For example, “creation of a therapeutic window” with reduced pain sensitivity through lumbar SMT, described by Louw et al. [36], may provide an opportunity for proper exercise and loading management ultimately improving self-efficacy with patients experiencing LBP, consistent with the suggestions of the most recent American Physical Therapy Association clinical practice guideline for LBP [2]. Therapeutic exercise and strength training have been shown to be beneficial for treatment of LBP [2, 37–39], particularly with the use of SMT for improving patient outcomes [2, 40, 41]. Understanding how these interventions coincide together in clinical practice may help to improve patient outcomes while empowering patients with strategies for improved self-efficacy.

We identified limitations in this study. First, PPT was only measured out to 30-minutes post-treatment. Although this may be the first trial to investigate the effects of lumbar SMT on PPT at 30-minutes post-treatment using a control group, further research is needed to investigate exactly the length this effect has beyond 30-minutes. Second, although we had a satisfactory sample size in an effort to reduce a type II error, the sample in this study were a homogenous group of younger, asymptomatic individuals. Future studies should include a larger age range of participants with LBP to improve more pragmatic participant designs for proper knowledge translation to the clinical setting. Third, although we blinded the participants to deactivated ultrasound parameters, each participant was aware of the other treatment group. Proper blinding of each intervention for all participants may help to eliminate potential bias for future studies. Fourth, we recognize the explanatory (efficacy) design of this study, posing challenges of knowledge translation to real-world clinical practice. The intent of this study was to reduce extraneous variables, but we recognize we encompassed a homogenous participant population, used a single intervention, and had no flexibility in the intervention delivery not typical of clinical practice. Fifth, although the outcome assessor was blinded to each intervention group, PPT was only measured once for each timepoint. We made this decision to reduce any skin sensitivity and potential soreness with multiple testing in one small testing region, but recognize this may have been skewed due to the participant’s unfamiliarity or hesitation with the technique. Lastly, this study only assessed at one location (ipsilateral right PSIS) following the lumbar SMT. While the findings from Dorron et al. [25] suggest that an increase in PPT is greatest at ipsilateral sites distal from the location of manipulation, assessing sites distal to the PSIS may be warranted for future research to better understand the neurophysiological effects of SMT.

## Conclusion

Lumbar SMT increases PPT at the ipsilateral PSIS immediately and 30-minutes following treatment in asymptomatic individuals compared to a deactivated ultrasound control group. The current data provides further insight into the mechanisms of SMT, particularly the temporal aspect of pain inhibitory mechanisms. Future studies should further explore beyond the immediate and short-term neurophysiological effects of lumbar SMT to validate these findings.

## Data Availability

All data produced or examined during this study are included in this manuscript. The data can be made available upon request.

## Abbreviations

SMT: Spinal manipulative therapy
LBP: Low back pain
PSIS: Posterior superior iliac spine
HVLAT: High-velocity low-amplitude thrust
PPT: Pain pressure threshold
IRB: Institutional review board
ANOVA: Analysis of variance
